# Clustering of Aromatic Amino Acid Residues around Methionine in Proteins

**DOI:** 10.3390/biom12010006

**Published:** 2021-12-21

**Authors:** Curtis A. Gibbs, David S. Weber, Jeffrey J. Warren

**Affiliations:** Department of Chemistry, Simon Fraser University, 8888 University Drive, Burnaby, BC V5A 1S6, Canada; cgibbs@sfu.ca (C.A.G.); dsw7@sfu.ca (D.S.W.)

**Keywords:** methionine, tyrosine, tryptophan, phenylalanine, non-covalent interactions, bioinformatics

## Abstract

Short-range, non-covalent interactions between amino acid residues determine protein structures and contribute to protein functions in diverse ways. The interactions of the thioether of methionine with the aromatic rings of tyrosine, tryptophan, and/or phenylalanine has long been discussed and such interactions are favorable on the order of 1–3 kcal mol^−1^. Here, we carry out a new bioinformatics survey of known protein structures where we assay the propensity of three aromatic residues to localize around the [-CH_2_-S-CH_3_] of methionine. We term these groups “3-bridge clusters”. A dataset consisting of 33,819 proteins with less than 90% sequence identity was analyzed and such clusters were found in 4093 structures (or 12% of the non-redundant dataset). All sub-classes of enzymes were represented. A 3D coordinate analysis shows that most aromatic groups localize near the CH_2_ and CH_3_ of methionine. Quantum chemical calculations support that the 3-bridge clusters involve a network of interactions that involve the Met-S, Met-CH_2_, Met-CH_3_, and the π systems of nearby aromatic amino acid residues. Selected examples of proposed functions of 3-bridge clusters are discussed.

## 1. Introduction

Noncovalent interactions, such as hydrogen bonding, ionic interactions (i.e., salt bridges), and the hydrophobic effect play many roles in the three-dimensional structure of a protein [1,2], interprotein interactions [3], and protein–ligand binding [4]. The intramolecular forces at play in proteins are of great interest, and the increase in submissions to the Protein Data Bank (PDB) has allowed for the details of these interactions to be systematically surveyed in a wide variety of macromolecules. Furthermore, new ideas have emerged surrounding aromatic amino acid residues and how they might affect protein structure and function. These include interactions such as π–stacking [5], cation–π [2,6], anion–π [7], and sulfur–aromatic interactions (S–π) [8]. Understanding this array of interactions is of great importance, and of great interest, for rationalizing protein structure and function.

**Figure 1:**
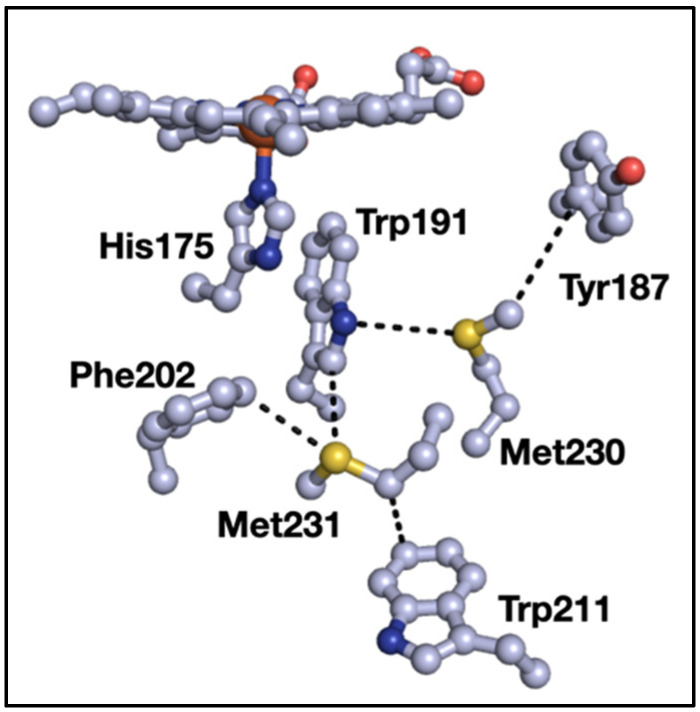
Examples of Met–aromatic interactions in yeast cytochrome *c* peroxidase (PDB ID 2CYP): Dashed lines indicate points of close contact and are for distance from 3.4 to 4.4 Å. Red = oxygen, blue = nitrogen, gray = carbon, yellow = sulfur. The backbone residues are omitted for clarity. The image was generated using PyMOL.

We previously surveyed sulfur–aromatic interactions in metalloproteins [9] and sulfur–aromatic interactions that involve two aromatic groups interacting with both lone pairs of sulfur [10]. During the course of those studies, we noticed a small but significant type of interaction where three aromatics clustered around the thioether of methionine (Met). At the time, we thought that these “3-bridge” clusters were outliers given the comparatively small size of sulfur (with respect to the aromatic groups), which we took as prohibitive in terms of interactions with three aromatic groups. However, ongoing work in our lab showed that such structures appeared in diverse proteins with different functions, and our focus solely on sulfur neglected other interactions of the Met-thioether. One such example is shown in Figure 1, where a bridging interaction exists between tryptophan 191 (Trp191), Met230, and tyrosine 187 (Tyr187) in yeast cytochrome *c* peroxidase (C*c*P) [11]. In addition, Met231 forms a bridging interaction with Trp191 and phenylalanine 202 (Phe202). Close inspection of the structure shows an interaction between the Met-CH_2_ and Trp211. Oxidation of either Met in the apo-protein alters the ability of the protein to make Compound I [12], and mutations alter the properties and stability of the Trp191 radical cation [13,14]. This example using C*c*P demonstrates some potential roles of Met–aromatic clusters in protein structure and function, which is the subject of this manuscript. Here, we provide a detailed analysis of the incidence and composition of 3-bridge clusters in structurally characterized proteins.

In general, the sulfur-containing amino acid residues, Met and cysteine (Cys), can stabilize protein microstructures through a noncovalent interactions that involve a Met or Cys sulfur and an aromatic ring [15]. It is known that alternating chains of sulfur-containing amino acid residues with Trp, Tyr, or Phe impact the structural properties of proteins and it is suggested that the redox properties of proteins may also be affected [15,16,17]. Small peptide models show a net stabilizing effect (ca. 1 kcal mol^−1^) of Met–aromatic interactions [18] and an extensive bioinformatics and computational analysis of membrane proteins show that several different Met–aromatic interactions are favorable [19]. In addition, bimolecular small molecule models of S–π interactions display advantageous enthalpies of formation of ~1 kcal mol^−1^ for the closed shell species [17,20]. S–π interactions also can confer unique redox and optical properties of small molecule models in their oxidized forms [20,21,22]. The distribution and properties of these interactions prompted us to investigate Met–aromatic clusters in greater detail, as described below.

## 2. Materials and Methods

### 2.1. Identification of “3-Bridge” Clusters in Protein Structures 

We first found closely spaced Met–aromatic groups meeting the criteria specified by our previously described [9,10] “Met–aromatic” algorithm (available at https://github.com/dsw7/MetAromatic (accessed on 20 September 2021) in a list of 33,819 protein X-ray structures with resolution better than 3 Å and sequence identity of less than 90%. Additional code and a list of structures and coordinates can be downloaded at: https://github.com/dsw7/n-Bridges (accessed on 20 September 2021). A file available as Supporting Information provides a list of PDB IDs, residues, and protein identities used for analysis. The dataset was collected from the Protein Data Bank [23] (PDB) in September 2019. The cutoff distance between Met and an aromatic residue was set to 6.0 Å and no cutoff angle was specified (i.e., approximated as 360°). The long cutoff distance was chosen such that weaker interactions could be detected. In our previous work [10], we treated closely spaced Met–aromatic residues as nodes in an imaginary network and then used the NetworkX library (https://networkx.github.io (accessed on 18 March 2020) to find 2-bridge interactions. Herein, a similar approach was used to find 3-bridge clusters, which are defined as Met residues with the faces of three aromatic groups (Phe, Tyr, or Trp) oriented in any geometry about the CH_2_-S-CH_3_ of Met.

### 2.2. Assessing the Position of Aromatic Residues about Methionine

To better understand how aromatic residues are positioned about Met, we examined the position of the various aromatic residues within the spherical region of space about the Met SD. Herein, we were interested in determining whether the aromatic residues assumed a preferential position relative to the Met CG-SD-CE scaffold. All 3-bridge clusters were assumed to consist of six 3-tuples: the CG-SD-CE (i.e., Met CH_2_-S-CH_3_) coordinates and three satellite points: C1, C2, and C3. The three satellite points were the centroids of the aromatic groups. All six 3-tuples were first mapped to the origin of a frame where SD assumed the origin (0, 0, 0 position). The system was temporarily isolated to CG-SD-CE and the direction cosine α between SD-CE and the *x*-axis was found. A Householder rotation of CG-SD-CE about the cross product of SD-CE and the *x*-axis by −α rendered the SD-CE bond axis colinear with the *x*-axis. The rotation was performed by a quaternion, Q1, subsequently rendering the CG coordinate free to rotate about the *x*-axis. Finally, the remaining CG coordinate was rendered coplanar with CE, SD, and the x,y-plane through rotation by angle −β about the *x*-axis and using a second quaternion, Q2. The aromatic centroids, C1, C2, and C3 were rotated into their final position using a composition of quaternions Q1 and Q2. All homogeneous transformations in this study were done with the assistance of the pyquaternion library (http://kieranwynn.github.io/pyquaternion/ (accessed on 18 March 2020)).

### 2.3. Density Functional Calculations

Calculations were carried out with the ORCA 4.2.1 ab initio quantum chemistry program [24,25]. The geometries of the hydrogens were optimized with the BP86 functional and def2-SVP/def2/J basis set [26,27] on all atoms. Single-point energy calculations used the BP86 functional and the def2-TZVP/def2/J basis set [26,27]. All calculations used the RIJCOSX algorithm and the Becke–Johnson damping scheme [28,29]. Natural bond orbital (NBO) [30] calculations were carried out using Gaussian16 (full citation in Supporting Information) using the BP86 functional and def2-TZVP basis set. Local Energy Dispersion calculations were carried out using DLNPO-CCSD(T) and the cc-pvtz and cc-pvdz basis sets [31]. Electron density plots were generated using UCSF-Chimera, developed by the Resource for Biocomputing, Visualization, and Informatics at the University of California, San Francisco, with support from NIH P41-GM103311 [32]. NBO plots were constructed using Avogadro [33].

## 3. Results

A total of 33,819 proteins were analyzed. The 3-bridge interaction was found 4751 times in 4093 unique structures (or 12% of the non-redundant dataset). A complete list of PDB codes and interacting amino acid residues are available in a separate file as Supporting Information. Most proteins showed one cluster, but there were examples of proteins with three or more 3-bridge clusters. About 48% of interactions were found in proteins without a stated Enzyme Classification (EC) number in their PDB entry; we note that 31% of PDB entries do not have an EC number attributed. The remaining 52% of hits are comprised of the following classifications, where the total percentage PDB-wide is given in parenthesis: 9.3% were EC 1 oxidoreductases (11% of PDB), 13% were EC 2 transferases (21% of PDB), 20.3% were EC 3 hydrolases (27% of PDB), 4.1% were EC 4 lyases (5% of PDB), 2.4% were EC 5 isomerases (2% of PDB), and 3.2% were EC 6 ligases (2% of PDB). No Met–aromatic clusters were observed for EC 7 translocases (1% of PDB). These data indicate that the Met–aromatic 3-bridge cluster is widely distributed in different classes of proteins and have about the same overall distribution as protein structures in the PDB.

**Figure 2:**
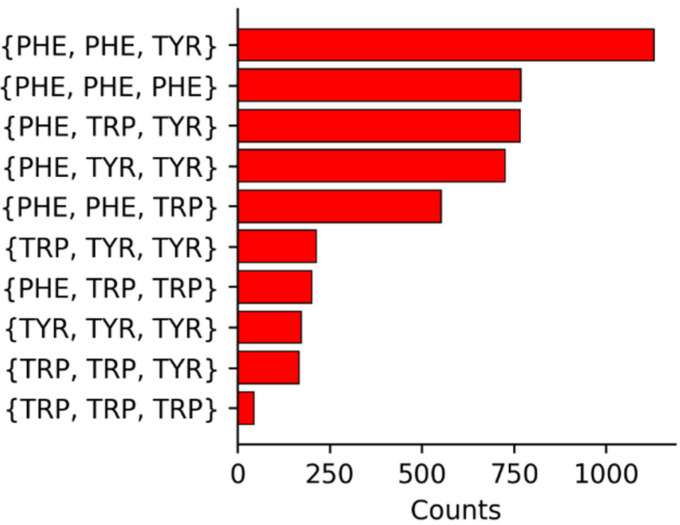
Summary of the identities and incidences of 3-bridge clusters.

The summary of the compositions of the three aromatic residues surrounding a given Met are shown in Figure 2. For reference, Phe has the highest occurrence in the UniProtKB/Swiss-Prot data bank of proteins [34] (3.9%), followed by Tyr (2.9%), and Trp (1.1%). In addition, Met occurs at a frequency of 2.4%. Given the relative incidences, the high occurrence of Phe as a member in 3-bridge clusters is not surprising. The higher counts for Trp in the 3-bridge clusters is interesting, given that Trp is found about one third as much as Phe and Tyr [34]. The overall larger degree of electron density on the face of the indole ring may promote stronger interactions with Met, providing a driving force for its relatively higher incidence.

**Figure 3:**
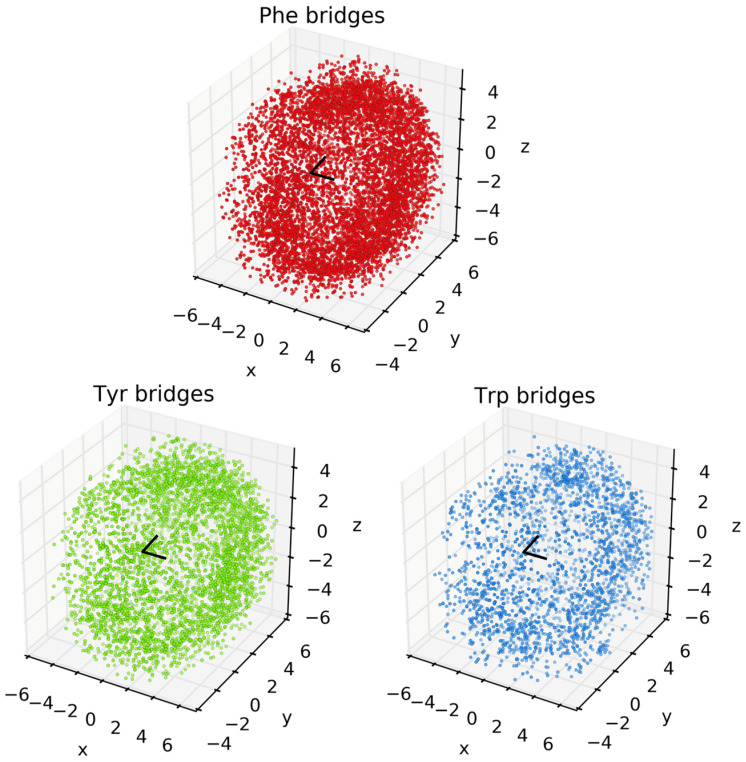
Plots of clustering of Phe, Tyr, and Trp around Met. The *x*, *y*, and *z* axes are in Ångstroms. The black V-shape at the origin depicts the CH_3_-S-CH_2_ thioether of Met. The arm pointing away from the reader (along +*y*) is the CH_2_ group. Each point corresponds to an aromatic centroid for each respective amino acid.

Analysis of the spatial orientations of the aromatic groups around Met are set out in Figure 3. The black V-shape at the center of the images denotes the CH_3_-S-CH_2_ thioether of Met. Each dot corresponds to an aromatic centroid. Note that this figure shows the raw incidences of Phe, Tyr, and Trp, regardless of the composition of the cluster. Images for the locations of aromatics in the different bridge compositions (i.e., Figure 2) are set out in the Supporting Information (see Figures S2–S11). While the search algorithm used a cutoff distance of 6 Å, the vast majority of hits occur at distances between 2 and 4 Å from the Met-CH_3_ or -CH_2_ to the aromatic ring. Note that the sum of the van der Waals radii of C and H is 2.9 Å. In all cases, the aromatic groups can be found widely distributed around the thioether, but there is a noticeably higher incidence of aromatics near the -CH_3_ and -CH_2_ groups (i.e., an interaction between a CH and a π system [35]). In some cases, this behavior is more apparent in isolated bridge compositions, for example, Phe, Trp, and Tyr clusters in Figure S4). While this work focuses on the incidences, basic features, and distance metrics of 3-bridge clusters, there also are angular correlations (e.g., between Met and faces of aromatics) that can be considered. This is the subject of our ongoing research.

**Figure 4:**
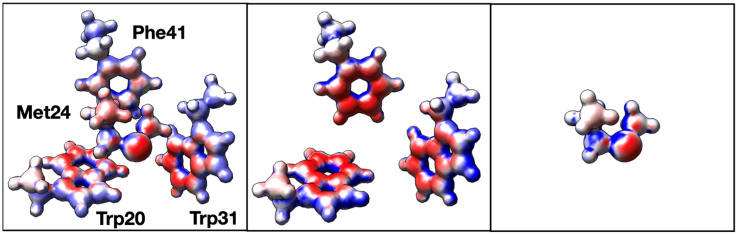
Electron density plots for the 3-bridge cluster in cytochrome P450 from *T. bispora* (PDB ID 5VWS). The (**left**) panel shows the complete bridge, the (**center**) shows the aromatic groups only, and the (**right**) panel shows Met only. Red corresponds to a charge of −0.03, white is zero, and blue is +0.03. The image was generated using UCSF Chimera.

In order to better understand the inter-residue forces that are at play in 3-bridge clusters, quantum chemical calculations were carried out using an example of a 3-bridge cluster from a cytochrome P450 found in *Thermobispora bispora* (PDB ID 5VWS) [36]. Hydrogens were placed programmatically in PyMOL [37]. Hydrogen positions were optimized, and electron density plots calculated, using the ORCA ab initio quantum chemistry program [24,25]. Three versions of the bridge cluster were initially explored (Figure 4): the complete bridge, the aromatic groups only, and the Met only. As expected, the faces of the aromatic groups have partial negative charges and the edges have partial positive charges. Likewise, the sulfur in Met has a partial negative charge. However, the partial charges are more pronounced in the 3-bridge components (middle and right panel of Figure 4) than in the whole cluster. While the effect is subtle, the decrease in the magnitude of the electron density is consistent with a weak dipole–dipole (van der Waals) interaction. The greatest change in electron density in the aromatics is observed in Trp20 and in Phe41, which we revisit below. Overall, this is an example of how the polarizability of the Met thioether and the delocalized aromatic systems may facilitate interactions in the 3-bridge clusters. Such dipole–dipole interactions have also been noted in detailed calculations of simple models of benzene and dimethyl sulfide [19].

**Figure 5:**
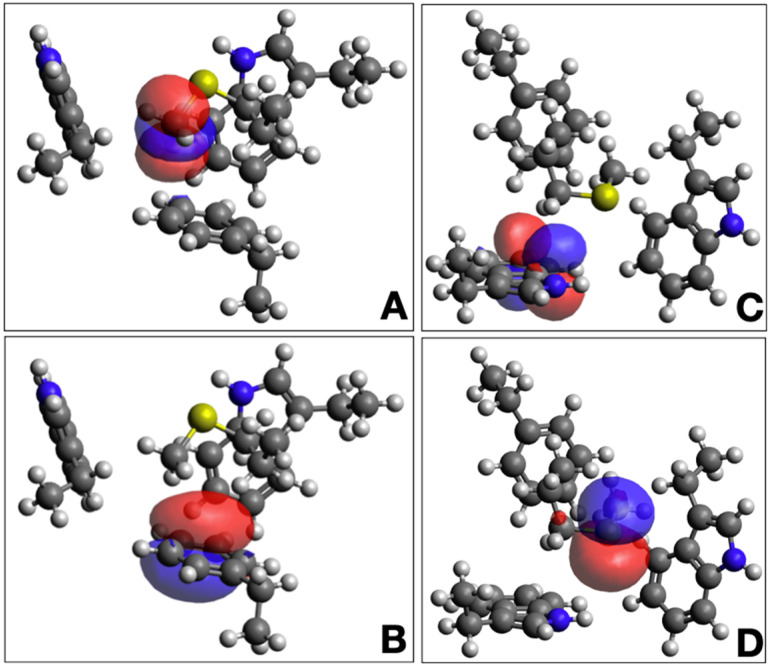
Natural bond orbitals from the 3-bridge cluster in cytochrome P450 from *T. bispora* (PDB ID 5VWS). Panels A and B show an interaction that involves a C-H* acceptor on Met24 (**A**) and a Phe41 C-C π donor (**B**). Panels C and D show an interaction that involves a Trp20 C-C π* acceptor (**C**) and a Met24 S lone pair donor (**D**). Gray atoms are carbon, white are hydrogen, blue are nitrogen, and yellow are sulfur. Blue and red orbitals represent positive and negative signs on the NBOs, respectively. The image was generated using Avogadro.

Additional insights can be gained through Natural Bond Orbital (NBO) analysis [30]. and energy decomposition using coupled-cluster (CCSD) calculations [31]. Again, we use the 3-bridge cluster shown in Figure 4 as an example case. The calculated interaction energy from CCSD calculations is −7.49 kcal mol^−1^, which is comprised of −7.73 kcal mol^−1^ of uncorrected interaction energy and 0.24 kcal mol^−1^ of geometric preparation energy [31]. The NBO calculation reveals that a complex network of orbital interactions is at play within the 3-bridge cluster. Two examples of contributing NBOs are shown in Figure 5. Interactions between all fragments can be observed, with the strongest interaction between Met24 and Phe41 and between Met24 and Trp20. The sum of all of the intermolecular interaction energies from the NBO second order perturbation theory analysis [E(2) values] is −6.4 kcal mol^−1^, consistent with the interaction energy from a higher level of theory.

In order to gain a more systematic view of how the interactions of aromatics affect the energetics of Met (and vice versa) in the context of the intact protein structure, the interaction energy matrix (IEM) web application [38] was used to evaluate interaction energies between Met and surrounding aromatic groups. Again, using the cytochrome P450 3-bridge cluster from Figure 4 (PDB ID 5VWS) as a model, a series of variants were programmatically produced where each member of the cluster amino acid residues was systematically replaced with alanine (Ala). Computations were carried out with the AMBER99 forcefield and using a water-like continuum, as set during submission in the IEM web application. Hydrogens were added programmatically by the web IEM application. The interaction energy values are set out in Table 1.

The largest values for pairwise interaction energies in the wild-type protein are observed for Met24-Trp20 and Met24-Phe41 pairs. This interaction energy also is roughly consistent with that obtained from NBO analysis (above). Consequently, replacement of either of those residues with Ala results in large changes in the net interaction energy of Met24 (shown in the first column of data). Interestingly, removal of either Trp results in a modest increase of the Met24-Phe41 pairwise interaction energy. The same is not true for removal of Phe41, in which case the pairwise interactions of Met24 with both Trp residues decrease. Replacement of all of the aromatics and Ala, or replacement of Met24 with Ala, results in a 75% decrease in the net energy of the residue at position 24. Analysis of a different bridge is shown in Table S1 (see Supporting Information), and similar conclusions can be drawn. This analysis suggests that interactions in 3-bridge clusters are complex and depend on the nature of individual amino acid residues.

## 4. Discussion

The 3-bridge interaction described here is a sub-class of Met–aromatic interactions that we [9,10] and others [15,19,38] described previously. In some ways, these interactions have features that resemble cation–π interactions [1,2]. They also can include aspects of CH–π interactions [35,39,40]. Such non-covalent interactions are known to play roles in structural biology. In cation–π interactions, the aromatic faces of Trp, Tyr, or Phe provide a negative electrostatic potential to allow for an interaction with a cation [39,40]. In proteins, a 1–5 kcal mol^−1^ increase in binding energy has been observed for these types of interactions, suggesting that they play roles in interprotein stabilization [1,6] and protein–ligand binding [39]. However, binding energies of over 20 kcal mol^−1^ are possible when a cation (e.g., lysine-NH_3_^+^) is surrounded by aromatics. Such a physical arrangement is similar to the Met–aromatic clusters described here. The energies of single cation–π interactions are only slightly more favorable than for single Met–aromatic interactions (1–3 kcal mol^−1^). Our results suggest that the 3-bridge clusters have interaction energies that are ca. 5–10 kcal mol^−1^ indicating that the cluster is favorable, but not as favorable as the analogous cation–π interaction. Finally, we note that cation–π interactions occur preferentially when the amino group is between 3.4 and 6.0 Å of the aromatic–π system [41]. These distance metrics are similar to the Met–aromatic clusters described here.

Similar to cation–π interactions, the CH–π interaction occurs between polarized CH and aromatic rings resulting in an attractive interaction that is dependent on amino acid conformation [42,43]. Consequently, this type if interaction is likely part of the overall Met–aromatic interaction. Different sub-types of CH–π interactions have been observed within protein structures, but Met most actively participates in the C_ali_H–π interaction (i.e., aliphatic CH donor) [35]. Overall, CH–π interactions could be part of the 3-bridge interactions, especially in those cases where favorable conformations between Met-CH and π acceptors are possible. In addition, the proximity and orientation of the aromatic residues in each 3-bridge cluster could give rise to C_aro_H–π interactions (i.e., aromatic CH donor). These interactions are thought to be stronger than their aliphatic counterparts [44].

The complete dataset for Met-aromatic 3-bridge clusters shows several proteins of the same, or similar, types. For example, iron superoxide dismutases (17 entries), DNA and RNA polymerases (38 entries), cytochromes P450 (28 entries), and chitinase (17 entries) enzymes appear in the dataset. These are intensely studied enzyme classes, so perhaps their high degree of representation is unsurprising. However, in all cases, the individual proteins show less than 90% sequence identity and can be found in different organisms. This suggests that the Met–aromatic clustering interaction is a general structural motif, rather than an isolated example in a single organism or class of proteins.

**Figure 6:**
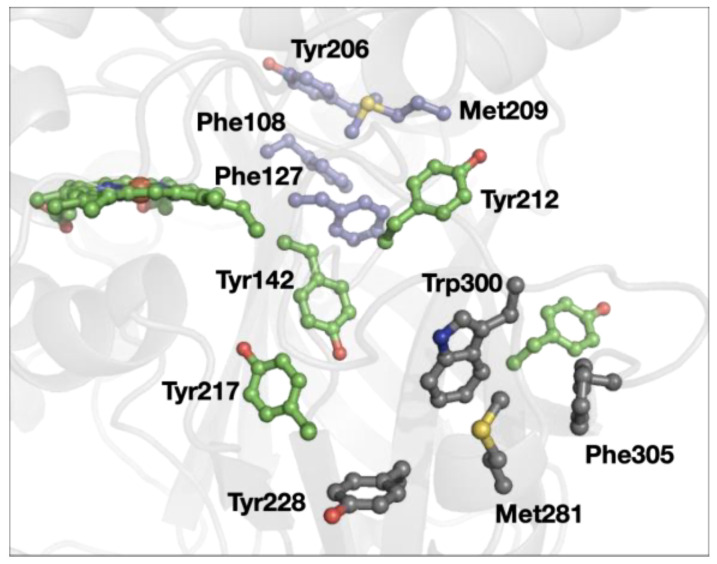
Structure of yeast catalase (PDB ID 1A4E [53]). The 3-bridge clusters are highlighted in gray and lavender, and the heme and other tyrosine residues are in green. Red corresponds to oxygen, yellow to sulfur, and blue to nitrogen. The image was generated using PyMOL.

A redox role of closely placed Tyr and Trp that has been proposed is the protection of redox-active proteins from off-cycle production of strong oxidants [45,46,47,48]. In some cases, the chains of Tyr and Trp can be both functional and protective, as in cytochrome *c* peroxidase (Figure 1) [49,50]. In our survey of 3-bridge clusters, we found examples of cases that could be part of protective Tyr/Trp pathways. For example, yeast catalase (Figure 6), Tyr228, Met281, Trp300, and Phe305 form a cluster near the surface of the protein. A series of Tyr (shown in green in Figure 6) connect the catalytic heme to the protein surface, with one potential pathway involving the 3-bridge cluster. Indeed, using Beratan’s pathway modeling tools [48], we find that Tyr228 is the favored hole acceptor (where the heme is the hole donor). The degree of electronic coupling between distant sites is an important determinant of electron/hole transfer rates, and such coupling is influenced by structural dynamics of electron/hole carriers [51,52]. In this context, the balance between stability and flexibility of Met–aromatic clusters may provide productive pathways for electron/hole flow in proteins.

Interestingly, yeast catalase has another 3-bridge cluster (Phe108, Phe127, Tyr206, and Met209, shown in lavender in Figure 6). This case provides an example of another common feature in the dataset: 3-bridge clusters that connect different parts of the protein (as evidenced by large separations in the primary structure). Again, the weak polar interaction of the Met and aromatics support the balance of stability and flexibility required for functional protein structures that is beyond a simple hydrophobic interaction.

**Figure 7:**
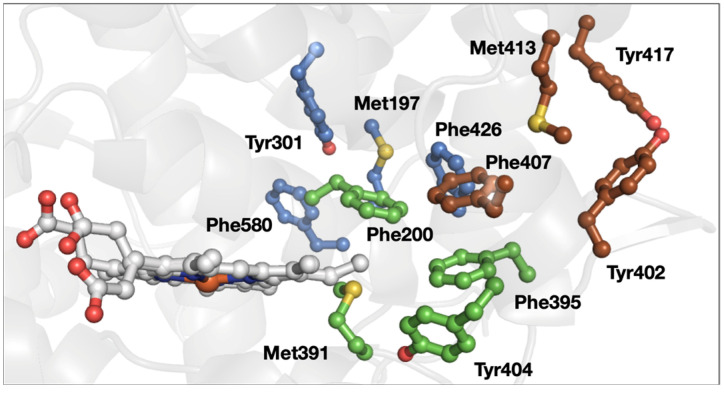
Structure of prostaglandin H2 synthase 1 (PDB ID 1Q4G [54]). The 3-bridge clusters are highlighted in maroon, green, and lavender, and the heme is shown in gray. Red corresponds to oxygen, yellow to sulfur, and blue to nitrogen. The image was generated using PyMOL.

One example of a protein that contains multiple 3-bridge clusters is prostaglandin H2 synthase 1 (e.g., PDB ID 1Q4G [54], Figure 7). Three different 3-bridge clusters localize between the heme and the protein surface. This is a particularly unusual example because of the close spatial proximity of the bridges in a medium-sized protein. Two Tyr residues (Tyr402 and Tyr417) are localized at the protein surface, making them strong candidates for a protective role [45,46,47,48]. The Tyr involved in the catalytic cyclooxygenase reaction (Tyr385 in this protein, not shown in Figure 7) is located toward the protein interior. The third bridge, involving Met197, Tyr301, Phe426, and Phe580, connects several different parts of the primary structure, which is consistent with a role in promoting tertiary structure and the enzyme active site via weak dipole–dipole interactions.

**Figure 8:**
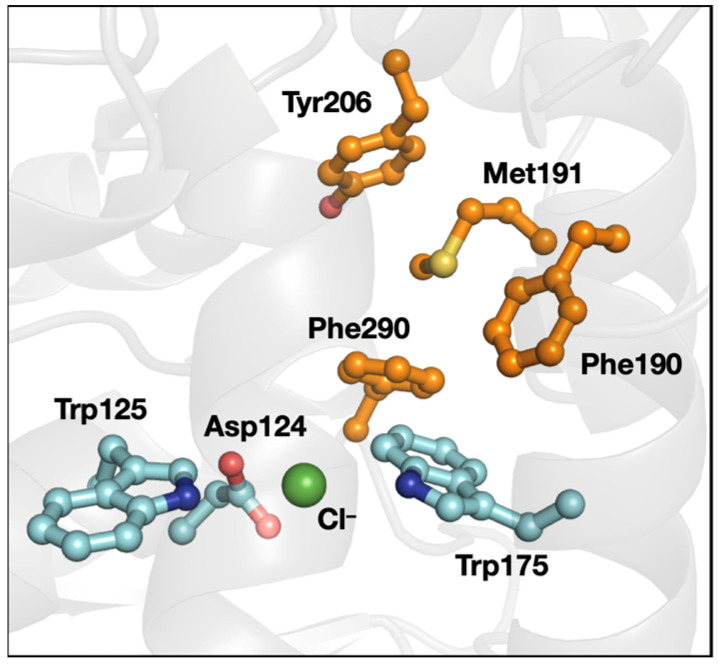
Structure of *X. autotrophicus* haloalkane dehalogenase (PDB ID 1B6G [55]). The 3-bridge cluster is highlighted in orange and the catalytic aspartate (Asp) and halide-stabilizing Trp are in cyan. Red corresponds to oxygen, yellow to sulfur, blue to nitrogen, and green to chloride. The image was generated using PyMOL.

As noted above, the 3-bridge clusters were found in all classes of enzymes, not just in oxidoreductases. *Xanthobacter autotrophicus* haloalkane dehalogenase catalyzes the dehalogenation of halogenated *n*-alkanes to generate the halide anions and corresponding alcohols. The chloride-bound X-ray structure of this protein (PDB ID 1B6G [55], Figure 8) shows the leaving halide stabilized by the indole rings of two Trp residues (Trp125 and Trp175). Halide loss is rate limiting during catalysis [56]. Trp175 is supported by two Phe residues (Phe190 and Phe290) that are part of a 3-bridge cluster. The motions of those Phe have been implicated in halide migration from the active site [57,58]. The dipole–dipole interactions that could be involved in the Met–aromatic 3-bridge cluster may have an impact on both maintaining local protein structure and enabling motions that promote loss of a charged halide product.

We propose that the intermediate energy of interaction in 3-bridge clusters can provide proteins with a balance of stability and flexibility. In the examples discussed above, B-factors can be investigated to evaluate the level of flexibility around a 3-bridge interaction. B-factors are commonly used when measuring the flexibility of protein–protein or protein–ligand binding sites [59,60]. In the examples provided above, 3-bridge clusters are within an intermediate range of flexibility with respect to the entire protein structure. Inspection of the individual B-factors suggests that a level of stability is imposed upon the protein structures around the 3-bridge clusters. A series of images that are colored to represent B-factors around each of the 3-bridge interaction sites are set out in the Supporting Information. Ultimately, the intermediate values of the B-factors suggest that a degree flexibility remains in those sites.

## 5. Conclusions

Structures where Met is surrounded by three aromatic groups are found in all types of proteins. In contrast to our previous surveys that focused solely on sulfur–aromatic groups [9,10], this work presents a more comprehensive perspective, where dipolar interactions of the entire Met thioether interacts with nearby aromatic groups. Inspection of selected examples show that Met–aromatic 3-bridge clusters could play roles in catalysis and in redox reactions. Analysis using computational methods suggest that clusters can be viewed, in some ways, as weaker analogs of cation–π interactions. The 3-bridges are characterized by a combination of interactions between the Met-sulfur, Met-CH_2_, and Met-CH_3_ groups, and the π systems of nearby aromatics. This weaker dipole–dipole interaction may strike an energetic balance between purely hydrophobic interactions and stronger cation–π moieties, allowing a level of protein flexibility while maintaining the protein’s native structure. Importantly, the entire Met-thioether is involved in 3-bridge clusters. The interaction is likely comprised by a combination of CH–π interactions, S–π interactions, and S-lone pair–π interactions. Overall, this represents a network of dispersive, electrostatic, and orbital interactions. To some extent, this contrasts with cation–π interactions, where the cationic group (e.g., Lys-NH_3_^+^) is the key component. Further studies of these Met–aromatic 3-bridge clusters, and other Met–aromatic interactions, will yield more insights on their properties and roles in protein structure and function.

## Figures and Tables

**Table 1:** Calculated interaction energies in the 3-bridge cluster in cytochrome P450 from *T. bispora* ^*a*^.

	Met24 *^b^*	Trp20 *^c^*	Trp31 *^c^*	Phe41 *^c^*
Wild Type	−10.9	−3.75	−2.02	−3.11
Trp20Ala	−7.44	−0.12	−2.01	−3.16
Trp31Ala	−9.12	−3.72	−0.31	−3.13
Phe41Ala	−7.54	−3.59	−1.87	−0.22
Trp20Ala/Trp31Ala	−4.21	−0.12	−1.86	−0.22
Trp20Ala/Phe41Ala	−5.68	−0.12	−0.31	−3.16
Trp31Ala/Phe41Ala	−5.98	−3.56	−0.37	−0.22
Trp20Ala/Trp31Ala/Phe41Ala	−2.68	−0.12	−0.37	−0.22
Met24Ala	−2.79	−0.65	−0.08	−1.23

*^a^* Energies in kcal mol^−1^. *^b^* Net interaction energy (side chain versus side chain). *^c^* Pairwise sidechain to sidechain interaction energy with the residue at position 24.

## Data Availability

The Met–aromatic algorithm is available at: https://github.com/dsw7/MetAromatic (accessed on 20 September 2021). Additional code and a list of structures and coordinates can be downloaded at: https://github.com/dsw7/n-Bridges (accessed on 20 September 2021).

## Supplementary Materials

The following are available online at https://www.mdpi.com/article/10.3390/biom12010006/s1, Figure S1: Summary of the identities of 3-bridges, Figures S2–S11: Plots of clustering of different aromatics around Met, Figures S12–S16: Color-coded residue flexibility using B-factors, Figure S17: Structure of biphenyl dioxygenase (corresponding to Table S1), Table S1: Calculated interaction energies in the 3-bridge cluster biphenyl dioxygenase from *C. testosterone*.
