# Supplementary material for: Clustering of Aromatic Amino Acid Residues around Methionine in Proteins
Source: Biomolecules. 2021 Dec 21;12(1):6. doi: 10.3390/biom12010006 (PMC8774105; doi:10.3390/biom12010006)
Supplement: Supplementary file 1 [file biomolecules-12-00006-s001.zip › biomolecules-1471193-supplementary/biomolecules-1471193_supporting/biomolecules-1471193_supporting.pdf]

Supporting Information for:

Clustering of aromatic amino acid residues around methionine in proteins

Curtis A. Gibbs,[†] David S. Weber,[†] and Jeffrey J. Warren*

Simon Fraser University
Department of Chemistry
8888 University Drive
Burnaby BC V5A 1S6
Canada

*jjwarren@sfu.ca

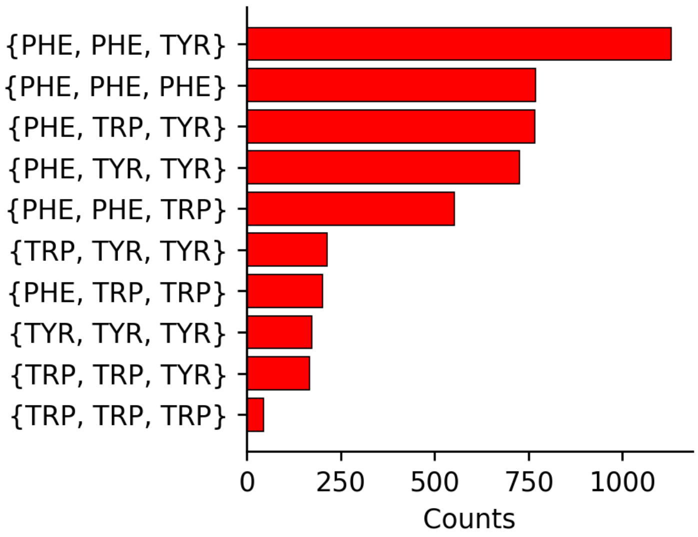

Figure S1. Summary of the identities of 3-bridges. The counts correlate with the number of points shown in Figures S2 to S11, below.

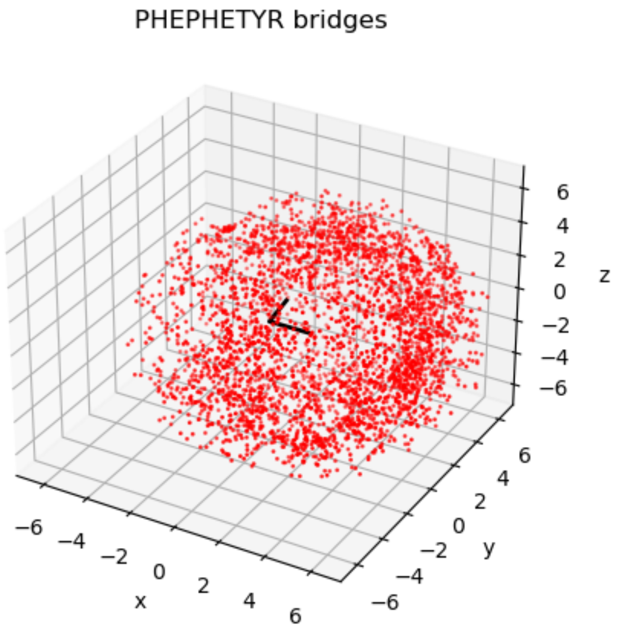

Figure S2. Plots of clustering of Phe and Tyr around Met in {Phe, Phe, Tyr} bridges. The x, y, and z axes are in Ångstroms. The black V-shape at the origin depicts the CH_3-S-CH_2 thioether of Met. The arm pointing away from the reader (along +y) is the CH_2 group. Each point corresponds to an aromatic centroid for each respective amino acid residue.

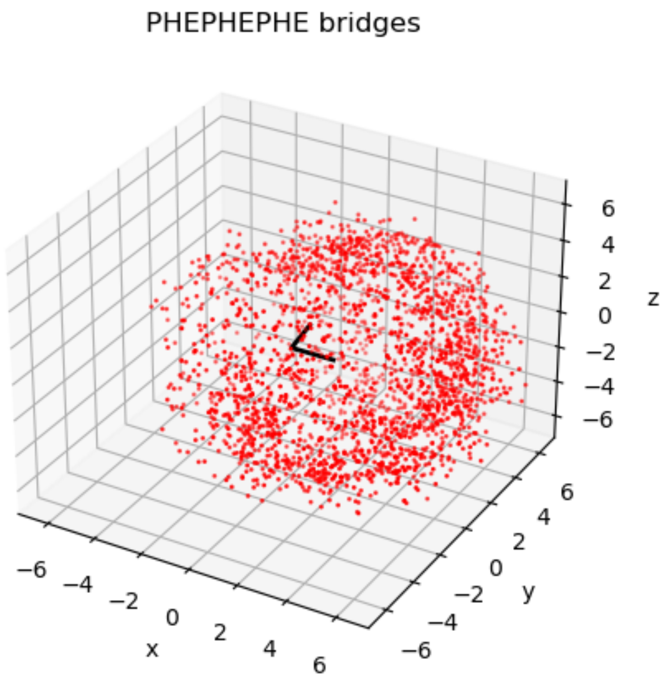

Figure S3. Plots of clustering of Phe around Met in {Phe, Phe, Phe} bridges. The x, y, and z axes are in Ångstroms. The black V-shape at the origin depicts the CH_3-S-CH_2 thioether of Met. The arm pointing away from the reader (along +y) is the CH_2 group. Each point corresponds to an aromatic centroid for each respective amino acid residue.

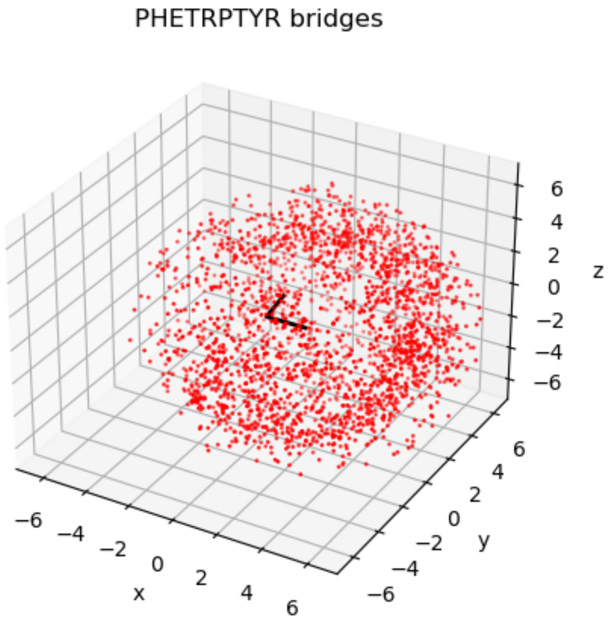

Figure S4. Plots of clustering of Phe, Trp, and Tyr around Met in {Phe, Trp, Tyr} bridges. The x, y, and z axes are in Ångstroms. The black V-shape at the origin depicts the CH_3-S-CH_2 thioether of Met. The arm pointing away from the reader (along +y) is the CH_2 group. Each point corresponds to an aromatic centroid for each respective amino acid residue.

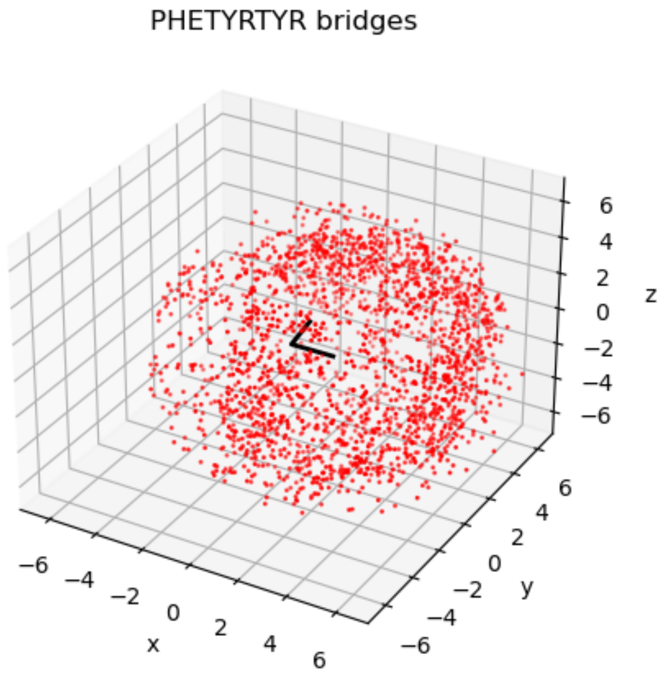

Figure S5. Plots of clustering of Phe, Tyr, and Tyr around Met in {Phe, Tyr, Tyr} bridges. The x, y, and z axes are in Ångstroms. The black V-shape at the origin depicts the CH_3-S-CH_2 thioether of Met. The arm pointing away from the reader (along +y) is the CH_2 group. Each point corresponds to an aromatic centroid for each respective amino acid residue.

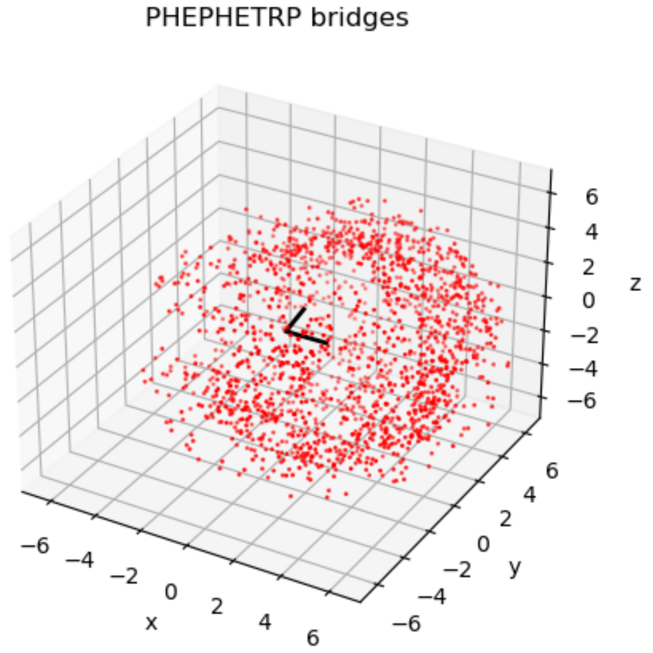

Figure S6. Plots of clustering of Phe, Phe, and Trp around Met in {Phe, Phe, Trp} bridges. The x, y, and z axes are in Ångstroms. The black V-shape at the origin depicts the CH_3-S-CH_2 thioether of Met. The arm pointing away from the reader (along +y) is the CH_2 group. Each point corresponds to an aromatic centroid for each respective amino acid residue.

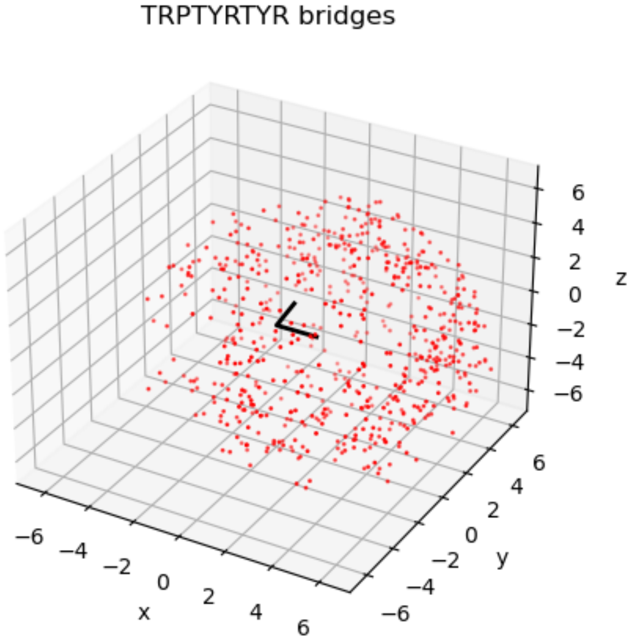

Figure S7. Plots of clustering of Trp, Tyr, and Tyr around Met in {Trp, Tyr, Tyr} bridges. The x, y, and z axes are in Ångstroms. The black V-shape at the origin depicts the CH_3-S-CH_2 thioether of Met. The arm pointing away from the reader (along +y) is the CH_2 group. Each point corresponds to an aromatic centroid for each respective amino acid residue.

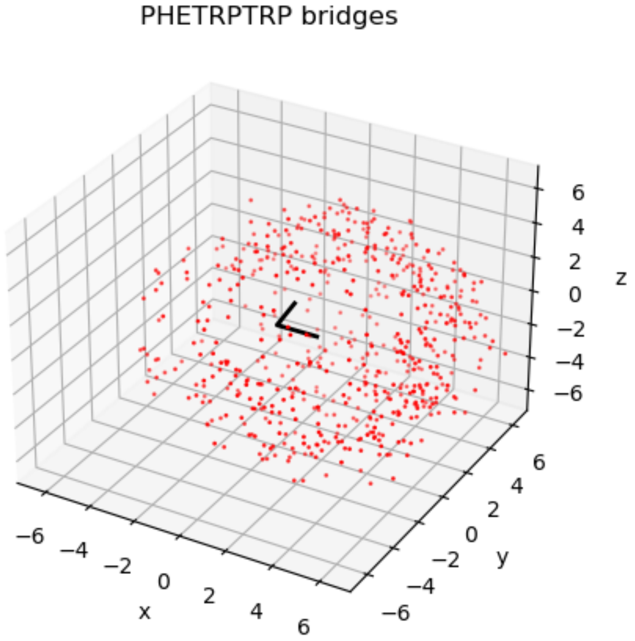

Figure S8. Plots of clustering of Phe, Trp, and Trp around Met in {Phe, Trp, Trp} bridges. The x, y, and z axes are in Ångstroms. The black V-shape at the origin depicts the CH_3-S-CH_2 thioether of Met. The arm pointing away from the reader (along +y) is the CH_2 group. Each point corresponds to an aromatic centroid for each respective amino acid residue.

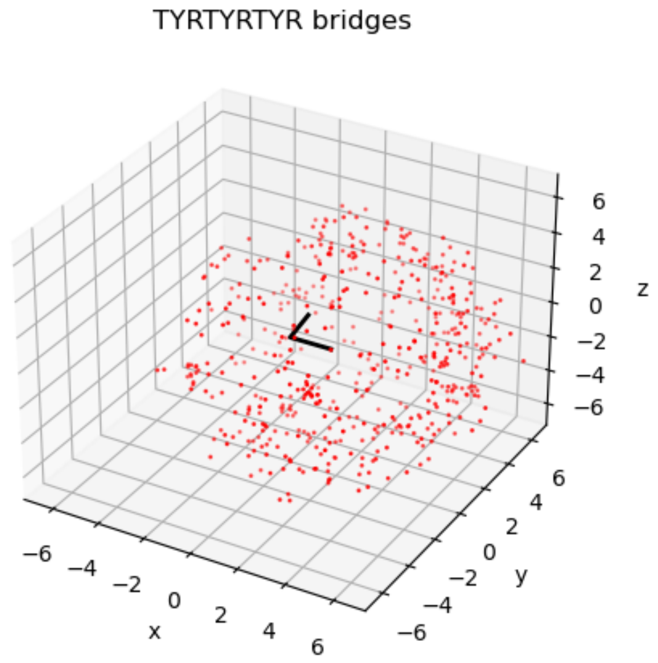

Figure S9. Plots of clustering of Tyr, Tyr, and Tyr around Met in {Tyr, Tyr, Tyr} bridges. The x, y, and z axes are in Ångstroms. The black V-shape at the origin depicts the CH_3-S-CH_2 thioether of Met. The arm pointing away from the reader (along +y) is the CH_2 group. Each point corresponds to an aromatic centroid for each respective amino acid residue.

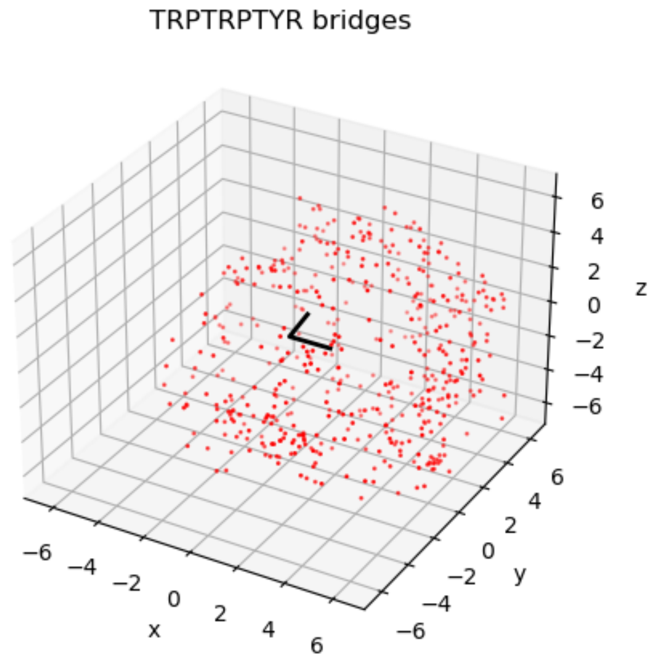

Figure S10. Plots of clustering of Trp, Trp, and Tyr around Met in {Trp, Trp, Tyr} bridges. The x, y, and z axes are in Ångstroms. The black V-shape at the origin depicts the CH_3-S-CH_2 thioether of Met. The arm pointing away from the reader (along +y) is the CH_2 group. Each point corresponds to an aromatic centroid for each respective amino acid residue.

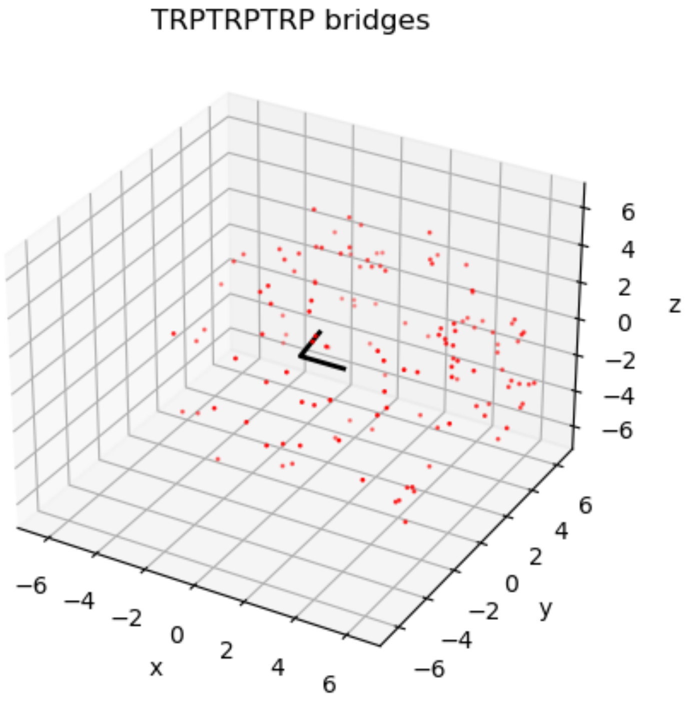

Figure S11. Plots of clustering of Trp, Trp, and Trp around Met in {Trp, Trp, Trp} bridges. The x, y, and z axes are in Ångstroms. The black V-shape at the origin depicts the CH_3-S-CH_2 thioether of Met. The arm pointing away from the reader (along +y) is the CH_2 group. Each point corresponds to an aromatic centroid for each respective amino acid residue.

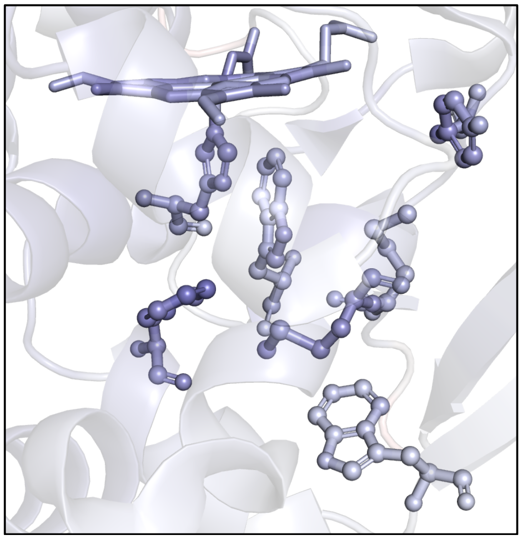

Figure S12. Flexibility color spectrum for yeast cytochrome *c* peroxidase (PDB ID 2CYP) using residue B-factors from the PDB. The spectrum from dark purple through white to dark red represents lower to higher areas of flexibility, respectively.

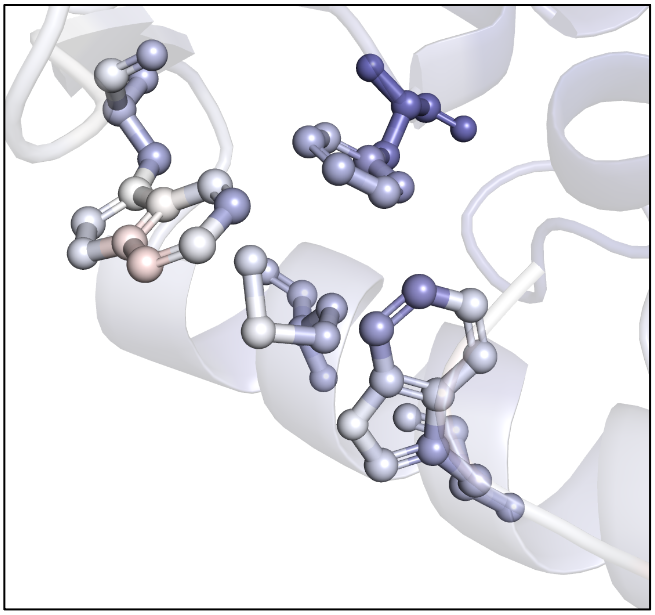

Figure S13. Flexibility color spectrum for cytochrome P450 from *T. bispora* (PDB ID 5VWS) using residue B-factors from the PDB. The spectrum from dark purple through white to dark red represents lower to higher areas of flexibility, respectively.

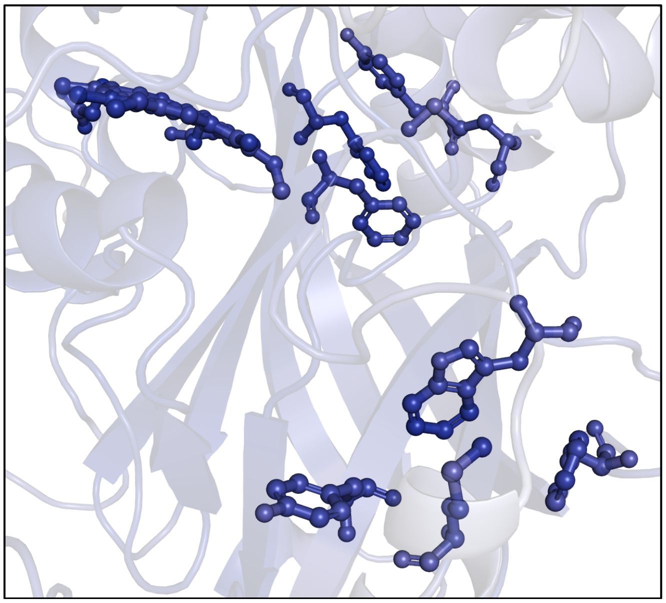

Figure S14. Flexibility color spectrum for yeast catalase (PDB ID 1A4E) using residue B-factors from the PDB. The spectrum from dark purple through white to dark red represents lower to higher areas of flexibility, respectively.

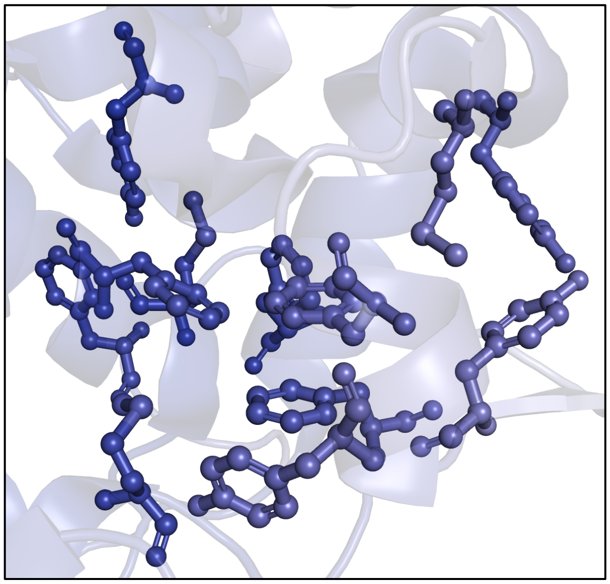

Figure S15. Flexibility color spectrum for prostaglandin H2 synthase 1 (PDB ID 1Q4G) using residue B-factors from the PDB. The spectrum from dark purple through white to dark red represents lower to higher areas of flexibility, respectively.

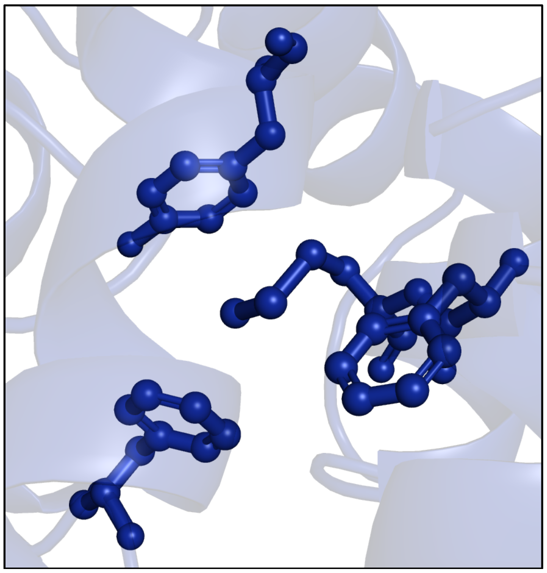

Figure S16. Flexibility color spectrum for haloalkane dehalogenase from *X. autotrophicus* (PDB ID 1B6G) using residue B-factors from the PDB. The spectrum from dark purple through white to dark red represents lower to higher areas of flexibility, respectively.

Table S1. Calculated interaction energies in the 3-bridge cluster in biphenyl dioxygenase from *C. testosterone* (PDB ID 3GZY)[a]

	Met439 [b]	Trp220[c]	Phe325 [c]	Tyr440 [c]
Wild type	-8.71	-0.60	-0.93	-2.29
Trp220Ala	-8.16	-0.01	-0.93	-2.28
Phe352Ala	-7.71	-0.58	-0.12	-2.28
Tyr440Ala	-6.55	-0.55	-0.92	-0.20
Trp220Ala/Phe325Ala	-7.18	-0.01	-0.12	-2.27
Trp220Ala/Tyr440Ala	-6.07	-0.01	-0.87	-0.20
Phe325Ala /Tyr440Ala	-5.58	-0.52	-0.12	-0.20
Trp220Ala/Phe325Ala/Tyr440Ala	-5.13	-0.01	-0.12	-0.20
Met439Ala	-1.52	-0.01	-0.20	-0.37

[a] Energies in kcal mol^{-1}. [b] Net interaction energy (side chain versus side chain). [c] Pairwise sidechain to sidechain interaction energy with the residue at position 439.

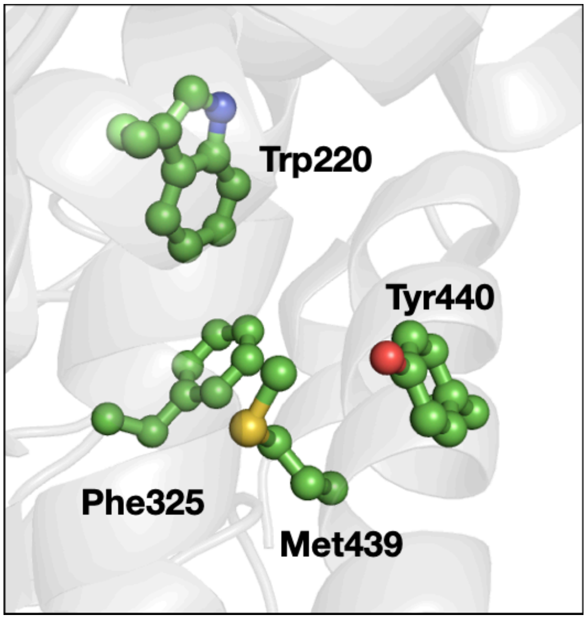

Figure S17. Structure of the 3-brdige cluster in biphenyl dioxygenase from *C. testosterone* (PDB ID 3GZY). The structure corresponds to Table S1.
